# Coping with moral distress on acute psychiatric wards: A qualitative study

**DOI:** 10.1177/09697330211010246

**Published:** 2021-09-06

**Authors:** Trine-Lise Jansen, Marit Helene Hem, Lars Johan Danbolt, Ingrid Hanssen

**Affiliations:** Lovisenberg diakonale høgskole (Lovisenberg Diaconal University College), Norway; MF Norwegian School of Theology, Religion and Society, Norway; 87368 VID Specialized University, Norway; MF Norwegian School of Theology, Religion and Society, Norway; 155319 Lovisenberg diakonale høgskole (Lovisenberg Diaconal University College), Norway

**Keywords:** Acute psychiatry, coping, mental health nurses, moral distress, nurses

## Abstract

**Background:**

Nurses working within acute psychiatric settings often face multifaceted moral dilemmas and incompatible demands.

**Methods:**

Qualitative individual and focus group interviews were conducted.

**Ethical considerations:**

Approval was received from the Norwegian Social Science Data Services. Ethical Research Guidelines were followed.

**Participants and research context:**

Thirty nurses working within acute psychiatric wards in two mental health hospitals.

**Results:**

Various coping strategies were used: mentally sorting through their ethical dilemmas or bringing them to the leadership, not ‘bringing problems home’ after work or loyally doing as told and trying to make oneself immune. Colleagues and work climate were important for choice of coping strategies.

**Discussion:**

Nurses’ coping strategies may influence both their clinical practice and their private life. Not facing their moral distress seemed to come at a high price.

**Conclusions:**

It seems essential for nurses working in acute psychiatric settings to come to terms with distressing events and identify and address the moral issues they face. As moral distress to a great extent is an organisational problem experienced at a personal level, it is important that a work climate is developed that is open for ethical discussions and nourishes adaptive coping strategies and moral resilience.

## Introduction

Nurses working within acute psychiatric settings – that is, giving treatment and care during an acute phase of mental illness – often find themselves in situations facing multifaceted moral dilemmas and incompatible demands. This may cause moral distress.^1^ Moral distress is an increasingly familiar term and a common phenomenon in many healthcare contexts and professional groups.^2^ The concept is attributed to Jameton^3^ and may be defined as an unpleasant feeling or a psychological imbalance which arises when one knows what the ethically right action is, but internal and/or external factors make it impossible to act accordingly. Moral distress may also arise when caregivers face moral dilemmas or experience moral doubt.^1,2,4^

Moral distress may be a positive reminder of moral obligation, keep us alert to moral dilemmas and help us maintain high standards of care.^5,6^ However, moral distress tends to affect negatively both the quality of healthcare delivery and the well-being of the healthcarers themselves.^7^ Unresolved moral distress may lead to feelings of guilt, bad conscience, sadness, powerlessness, emotional numbness, shame, cynicism, despondency, anger, angst, self-criticism and resignation.^1,8–10^ It may violate one’s integrity^8^ and produce personal and professional disillusionment.^10^ Nurses who experience moral distress tend to withdraw emotionally from patients^9,11^ and disconnect from themselves and others.^9,12^ Common physical symptoms are fatigue, exhaustion, headaches, stomach pain, sleeplessness, weight changes and palpitations.^8,9,13^ Thus moral distress may cause staff turnover,^1,10,11,14,15^ burnout^10,13,15,16^ and is ultimately harmful to patients.^9,10,15,16^

Frequently experienced morally stressful situations may cause moral residue, ‘that which each of us carries with us from those times in our lives when in the face of moral distress we have seriously compromised ourselves or allowed ourselves to be compromised’,^17^ resulting in increased levels of moral distress, the so-called crescendo effect.^18^ Rushton et al.^19^ claim that ‘few solutions have been proposed for alleviating a problem that is only expected to escalate as healthcare becomes more complex’ (p. 82). In line with Rushton et al.’s findings, various interviews with mental healthcare workers indicate that the challenges within acute psychiatric care are escalating.^1,20^ Increase in challenges may increase moral distress.

How nurses in acute psychiatric care cope when repeatedly being exposed to moral distress in their clinical practice is sparsely investigated. Rushton^8^ points to moral resilience as a defence against moral distress, that is, ‘the capacity of an individual to sustain or restore [her or his] integrity in response to moral complexity, confusion, distress, or setbacks’. Exploring ways of coping can lead to the knowledge necessary to understand what kinds of support, skills, structure and so on nurses need to find strategies that can mitigate the negative effects of moral distress. Staff in mental care settings report poorer well-being and a higher absence rate than in other healthcare sectors.^21^ It is clear that burnout is a significant problem in mental health.^22^ Morse et al.^22^ comment on the irony of ‘the mental health field [having] paid relatively little attention to the health and well-being of its own workers’ (p. 10).

Molewijk et al.^23^ have ‘found little information on how health care professionals actually deal with ethical challenges in health care’ (p. 2). How to ‘deal with’ present and future legislation was very much on the interviewees’ minds. To cope means to ‘carry on, get by, make do, manage, survive’.^24^ In this article, the question of ‘dealing with’ moral distress is seen in light of coping. The aim is to explore how nurses attempt to cope when in moral distress. Our research question is as follows:

*RQ.* How do nurses working in acute psychiatric settings cope with moral distress?

## Methods

A qualitative design with in-depth interviews and focus group interviews was chosen to acquire insights into the interviewees’ subjective experiences, attitudes and thoughts^25,26^ concerning coping with moral distress. Qualitative research is well suited to study the complexities of this phenomenon and how moral agents experience moral distress in dynamic contexts.

This article is part of a larger study on sources, features and reactions to moral distress in nurses working in acute psychiatric settings.^1^ Questions concerning coping were among the themes discussed. In-depth individual interviews were conducted with a total of 16 nurses in two different mental health hospitals. In addition, we did three focus group interviews with a total of 14 nurses working on acute psychiatric wards (Table 1). In these groups, the focal point was the sharing of common experiences and associations rather than group dynamics. This created somewhat different data and ideas than the individual interviews did.^27^ In all the focus group interviews and about 50% of the individual interviews, two interviewers/moderators were present.

**Table 1.** Background and number of nurses interviewed.

Years of psychiatric nurse experience	Individual interviews	Focus group interviews	No. of mental health/psychiatric nurse specialists among the interviewees
0–5	2	4	
5–10	6	6	8
10–15	2		2
15–20	2	2	2
20+	4	2	6

A purposive sampling strategy was used to identify potential participants. These were identified by the heads of the acute psychiatric units in question. These were all informed orally and in writing about the study’s content, purpose and goal.

Inclusion criteria are registered nurses with work experience in the field. The interviews took form of electronically recorded talks where the participants were encouraged to share their thoughts and recount challenging experiences. Follow-up questions and the ‘mirroring’ of statements were used to develop, clarify and verify statements.

### Data analysis

The first author, a psychiatric nurse specialist, transcribed the interviews verbatim. Two of the co-authors, both nurse ethicists, took part in the data collection. All authors participated in the data analysis. All the interview texts were analysed together in light of coping with moral distress. We used Braun and Clarke’s^28^ six analytic phases for thematic analysis: (1) the authors familiarised themselves with the interview data through reading and re-reading the interview texts. (2) Interesting features were coded and (3) collated into potential themes.^28^ Phases 4 (reviewing themes) and 5 (defining and naming themes) were done collaboratively by all the authors. We kept returning to the transcripts to ensure that our interpretations were supported by the data. (6) The first author’s preliminary text was discussed and developed further collaboratively.

Analytic credibility is obtained through quotations with interviewees’ own description of thoughts and experiences. Rigour is obtained through being four analysts. The researchers having different professional backgrounds, two with an insider view as psychiatric nurses and two from other fields of expertise, add value to the analysis.

### Ethical considerations

Approval was given by the Norwegian Centre for Research Data. All interviewees were informed orally and in writing that participation was confidential and voluntary, and that they were free to withdraw from the project. All signed an informed consent form. Interview transcriptions are stored safely according to Ethical Research Guidelines.^29^ Recorded interviews were deleted after transcription.

### Strengths and limitations

The majority of the interviewees were mental health specialists with many years’ experience from acute psychiatric care (Table 1). As participation was voluntary, we cannot say whether the views presented are representative for all nurses in the hospital units in question. Although our study is fairly local, we believe the insights offered are transferable to other acute care psychiatric nursing contexts and thus may help decrease the current paucity of knowledge within this field.

## Results

To care for persons who suffered greatly mentally was seen as meaningful and gratifying despite the fact that their work tended to influence their private life and even their well-being:

This is what keeps you going, that you feel that you can do a lot of good for so many…although [they are] not totally cured, generally the patients are better on discharge. (N16)

However, in periods with inadequate staffing, and when work challenges and ethical dilemmas seemingly were piling up, some interviewees could find it necessary to take a day off or even go on sick leave. They realised that they were unable to do a good job without a respite when physically and mentally exhausted: ‘You work with problems all the time; you cannot have problems yourself. You have to be rested and fresh’ (N12).

The interviewees’ coping mechanisms may be divided into three main themes: through ‘sorting’ their thoughts and feelings, not taking work home with them or loyalty versus speaking up.

### Sorting thoughts and feelings

Lack of resources that frustrated the nurses’ ability to give patients the care they needed was often mentioned. One of the nurses had furthermore witnessed ‘unjustifiable’ conduct that could escalate into dangerous situations both from unskilled extras and professional staff. The importance of ‘sorting’ their thoughts and feelings was discussed by many of our interviewees. They found this ‘sorting work’ as one of them called it essential both for being able to process and cope with their experiences and for finding their work meaningful and worthwhile over time, despite dilemmas and worry about the quality of treatment and care.

The need to work through their thoughts and feelings was handled differently on the different wards. It seemed particularly focused on one of the wards. There they talked about the positive effect of talking things through together:

To talk together and use each other’s experience and come to an agreement, that can remove stress. We are good at ventilating concerns, choices we have made. (N9)

N3 found that the

‘best way to cope is to talk with my colleagues about how I experience what has happened. If I am going to cope with this job for years, I need help to sort things through. If not, there is a great danger of burnout’. On her ward they discussed ethical dilemmas on a near daily basis. Even if something could have been done differently, it did not necessarily mean that it was done wrong. Therefore, it was important to ‘be able to talk about things afterwards’. (N9)

For some, discussing moral challenges with a pastor/priest or in a mentoring group was a helpful coping strategy. The latter was a setting where all kinds of themes could be discussed, and the nurses felt free to ‘talk about how it affects us’ (N3). Not every unit offered mentoring groups, though, and it was sorely missed by those who had previously taken part in such groups.

### Not taking their work home with them

The Norwegian concept of being ‘flink’ – being good at something; it is not quite translatable in this context – was much used among those who claimed that they did not think about work while at home, or ‘bring it home with them’, as they expressed it: ‘I do not bring anything home with me, absolutely not’ (N8). Even if there were a lot of things she disapproved of or saw as problematic at work, ‘I do not bother to bring it home with me’. N28 agreed and said that she had ‘learned to push it away when I get home’. Also, N6 claimed that she was quite ‘flink’ (good at) leaving her thoughts concerning work at work. Even so, some evenings, when in bed, she would think about certain episodes and wonder what she could have done differently. N2 held that ‘I have become quite flink/good at leaving it behind me on my way home, otherwise it would hardly be possible to work here’. Although N16 held that frustrations and ethical challenges at work did not affect her mood at home, she did at times worry that things at work influenced her private life.

Thus, there was an obvious dissonance between these nurses’ claim of not bringing their work home with them and what often occurred. Several interviewees admitted that although they tried not to mull over work when at home, they often felt ‘tired and grumpy when I get home and I need to sleep’ (N27.) And ‘while one really would have wanted to go for a run or be among friends, just have fun, one lacks the strength, becomes without initiative’ (N6). Others admitted that the thoughts ‘kind of pop up’ even when off duty because ‘there are some things that stick with you’.

N14 said she often felt frail, empty and tired when she got home from work, that she created a shield between herself and her surroundings and felt emotionally numb and that she was losing her role in her own life. Her way of coping was to fill her private life with good and beautiful things. N11 coped with feelings of work-related inner disquiet and inability to sleep by trying to make herself physically exhausted by working out, or periodically taking sleep medication. She also found that it helped to ‘stay in bed and binge on TV series or watch a bad movie’.

According to N13, years of experience as a psychiatric nurse had enabled her to ‘rapidly disengage from the many difficulties [at work] when I get home. But it worries me a little, too, that I have become cold and blasé’. Becoming emotionally numb, cold and distant was a worrying thought for several interviewees. N14 said that she tended to disengage her feelings when she was more tired than usual. As many patients were very perceptive, she felt this was unfortunate as they could comment on her seeming abstracted.

### Loyalty versus speaking up

Some interviewees coped with participating in treatment with which they disagreed by seeing themselves as loyal cogs in the machinery – as ‘part of the system’ – or by ‘just doing as [the physicians] have decided’. As N10 put it, ‘It is no problem for me that others have decided what I am to do, like giving coercive medication. I am no doctor, I cannot prescribe anything, I am to administer it’. Furthermore, ‘I can say that I will not give this, but then one of my colleagues will have to do it’ (N2). To participate in coercive treatments and to be exposed to violence were described as ‘inherent in our job’ by several interviewees.

The nurses clearly experienced and coped with these kinds of possible morally distressing situations differently. N9 found that when ‘it is difficult to know what is the best thing to do, your hands become clammy…that is a stressor’. Tension headache was another rampant bodily symptom. N5 tended to develop headaches on days when she knew they were short staffed, which meant that she would have to shoulder extra heavy responsibilities. She tended to feel guilty when patients became aggressive or violent and the safety of patients and/or nurses was threatened. This made her feel that she should have acted differently, it was her fault, and she should have prepared herself better. This kind of self-criticism – even guilt and shame – was often mentioned in connection with the use of coercion or inability to prevent aggressive behaviour and violence.

On one of the wards, there was room for critical questions concerning their clinical practice, and the Head Nurse listened to them and acknowledged their experiences. On this ward, ‘we are free to speak our minds. That helps’ (N27). Another pointed out that ‘if we should have kept it inside, we would have exploded a few times, I think, and not been able to stay on’. Freedom to be this candid about thoughts and feelings was not common on all the wards, though.

For some, the knowledge that they could quit their job somehow seemed to be an incentive to stay on. Several interviewees were seriously contemplating changing jobs. They had tried to speak up about problems like understaffing that could lead to inadequate patient care, but their complaints had neither been validated nor led to any change.

## Discussion

In this article, we discuss how nurses attempt to cope with difficult moral challenges both in their psychiatric practice and when off duty.

### Reappraising and seeking support from colleagues

Seeking support from colleagues has been identified as a common strategy for coping with moral distress.^14,30–33^ This is in line with some of our findings. Especially on one ward, the nurses reported that they habitually shared thoughts, feelings and experiences. On this ward, the nurses had worked an average of 12 years and most of them had post-bachelor specialty training. This might indicate that their combination of experience, education and trust in each other’s competency created the self-confidence needed to be honest and open with each other. These nurses’ experiences indicate that staff involvement in ethical discussions should be supported and promoted by the leadership.

Engaging in dialogue with colleagues can be seen as a form of reappraisal.^34^ Reappraisals may be among the most effective ways to cope with stressful situations as ‘we alter our emotions by constructing a new relational meaning of the stressfull encounter’ (p. 116).^34^ Many of our interviewees ‘alter [their] emotions’ and construct ‘a new rational meaning’ to orient themselves towards *caritas.*^35^ As a concept, caritas indicates the will to do good. Caring acts are coping strategies grounded in the nurses’ orientation towards caring.^35^ To do good may be an effective coping strategy as it may create compassion satisfaction.^36^ Perhaps this can be seen as existential coping.

However, discussing moral concerns may by some psychiatric nurses be perceived as either threatening to the participants or jeopardising team cohesiveness.^14^ Musto and Schreiber^37^ found that nurses with positive experiences from on-the-job dialogue may be able to accept that they have done their best, an acceptance that enables them to work with a renewed focus on the therapeutic relationship. Those with negative experiences from such dialogues were unable to accept that their work performance in morally difficult situations ‘is the best I can do’. This made them either leave the unit or talk about leaving.

Those of our interviewees who were thinking of quitting their jobs indicated having had negative experiences with dialogue in the workplace as their moral distress had been dismissed and silenced by H their unit Head. This illustrates the importance of fostering a positive ethical work climate where raising ethical questions is encouraged. This encouragement must come from the leadership who, if not in a position to address the morally challenging issues raised by staff members themselves, needs to provide resources which can facilitate ethics-related conversations.^19^ Organisational conditions and practices influence the way in which ethical issues and concerns are identified, discussed and decided,^38^ and engaging in dialogue may be the primary means for nurses to mentally work through the experience of moral distress.^37^ Thus, the more positive the ethical climate is perceived to be, the lower the reported moral distress, and vice versa.^9,39,40^

### Lack of control, but attempting to leave problems at work

Our interviewees often faced situations of moral distress in which they felt they had limited or no influence or control. While a strong sense of coping, or even mastery, reduces the risk for stress of conscience and protects against stress, ‘a low sense of mastery may evoke feelings of helplessness, possibly affecting the way the nursing staff experience ethical and moral dilemmas and thus increase the stress related to a troubled conscience’ (p. 15).^41^ This is supported by Ando and Kawano^31^ who hold that one of the reasons why psychiatric nurses fail to act in response to ethical problems is that some felt helpless while others felt gloomy and do not know how to cope with the problem. Of course, in some contexts, there may realistically speaking be few problem-solving possibilities.

Many nurses used compartmentalisation as a strategy to get on with their everyday life outside work. This was found also by Helmers et al.^42^ in their study, expressed as ‘shutting the door’. However, ‘there is no on-off button for emotions, they are in themselves autonomous’ (p. 31),^43^ and the advice often given to healthcarers about not taking the job home with them and on self-care may constitute an extra burden.^43^

In many of our interviewees, moral distress tended to surface as uneasiness, numbness and/or physical symptoms. Some even had nightmares. Taking sick leaves or going for a run may be effective short-term avoidance strategies to regain the strength needed to cope with work challenges in a healthy way. However, the body ‘tells tales’ and this kind of ‘self-care’ may become an added problem.

### Loyalty and make oneself immune as coping strategies

The interviews strongly indicate that nurses tend to be loyal and faithful to the system. Loyalty may stem from expectations from the workplace and its leadership and from the individual’s identity as a nurse,^44^ and loyalty is understood as a virtue. We found that loyalty also may be understood as a coping strategy, a way to disclaim responsibility, to mitigate moral distress. Through placing the responsibility on the leadership and on other professions, the nurses may abscond from their moral standards. This may be seen as an attempt to make oneself immune to moral conflicts one faces, a common coping strategy used by Irish psychiatric nurses.^14^ To be immune means to be invulnerable, proof, protected and unaffected.^24^ Our interviewees tried to achieve this by for instance arguing that other nurses would perform nursing actions if they themselves refused to do them. Others described feeling resigned, that they trivialised morally challenging situations or had distanced themselves from them, becoming more aloof, cold and blasé. Health and social workers who frequently experienced emotional dissonance, a discrepancy between felt and expressed emotions, reported higher levels of exhaustion, mental distress and absences from work.^45^

None of the nurses in the quantitative studies Oh and Gastmans^9^ reviewed reported positive strategies for coping with moral distress. Among the mentioned strategies were leaving or considering leaving their job, as also seen in our study. However, ‘[s]ome nurses may become accustomed to moral distress as they gain experience, and some may suffer from cumulative moral distress’ (p. 27).^9^ It is therefore important that ‘[h]ealth care workers can learn to respond positively to ethically challenging situations by building their capacity for moral resilience, and organizations can support them by creating a culture of ethical practice’ (p. 82).^19^

## Conclusion

Our interviewees reported on various coping strategies. For some, sorting through the ethical dilemmas they experienced seemed to lead to moral resilience, while others tried to solve problems by bringing them to the leadership.

None of those who sought to ‘leave’ their problems ‘at work’ seemed to succeed in doing so. Rather, not facing their moral distress seemed to come at a high price. And, loyalty as a coping mechanism might become a source of moral distress rather than a distinguisher. Thus, how nurses cope with moral distress may influence both their clinical practice and their private life. It seems essential for nurses working on acute psychiatric wards to come to terms with distressing events and identify and address the moral issues they face. Independent of coping strategies, caritas seems to be a driving force.

Moral distress is to a great extent an organisational problem, albeit experienced at a personal level. It is important for unit leadership to foster a climate for ethical discussions and reappraisals of experiences and treatment choices. More research is needed regarding what promotes adaptive coping strategies and moral resilience among nurses in the complex field of acute psychiatric care.
